# Potent antibacterial activity of MSI-1 derived from the magainin 2 peptide against drug-resistant bacteria

**DOI:** 10.7150/thno.39157

**Published:** 2020-01-01

**Authors:** Lingman Ma, Xin Xie, Hanhan Liu, Ya Huang, Haomin Wu, Meiling Jiang, Pengfei Xu, Xinyue Ye, Changlin Zhou

**Affiliations:** School of Life Science and Technology, China Pharmaceutical University, Nanjing, Jiangsu 211198, PR China

**Keywords:** AMPs structural modification, MSI-1, antibacterial activity, drug-resistant bacteria, membrane rupture, DNA binding

## Abstract

The structural modification of existing AMPs is an effective strategy to develop antimicrobial agents with high-efficiency, low-cost and low-toxicity antimicrobial agents.

**Methods**: Here, we truncated 14-amino-acids at the N-terminus of MSI-78 to obtain MSI and further modified MSI to obtain four peptide analogs: MSI-1, MSI-2, MSI-3 and MSI-4. These peptide mutants were evaluated regarding their antibacterial activity against various sensitive or resistant bacteria; toxicity against mammalian cells or mice; and stability against violent pH, temperature variations and high NaCl concentrations. Finally, we also elucidated the possible mechanisms underlying its mode of action.

**Results**: The results showed that MSI-1 and MSI-3 displayed activity that was superior to that of MSI-78 with MICs of 4-16 μg/ml and MBCs of 8-64 μg/ml, respectively, especially against drug-resistant bacteria, due to the increase in percent helicity and amphiphilicity. However, MSI-3, with higher hydrophobicity and antibacterial activity, had a relatively higher hemolysis rate and toxicity than MSI-1. MSI-1 exerted rapid bactericidal activity and effectively improved the survival rate and wound closure in penicillin-resistant *E. coli*-infected mice by eliminating bacterial counts in mouse organs or subeschar, further inhibiting the systemic dissemination of bacteria. Additionally, MSI-1 displayed perfect stability against violent pH, temperature variations and high NaCl concentrations and has the ability to circumvent the development of drug resistance. In terms of the mode of action, we found that at the super-MIC level, MSI-1 exhibited direct antimicrobial activity by disrupting the integrity of the bacterial cell membrane, while at the sub-MIC level, it bound to bacterial DNA to inhibit DNA replication and protein expression and ultimately disrupted bacterial biological function.

**Conclusions**: This novel peptide MSI-1 could be a potential candidate for drug development against infection induced by drug-resistant bacteria.

## Introduction

The increasing emergency to combat the rapidly widespread resistant bacteria has prompted the development of new generations of antimicrobial agents, with AMPs being one of the most promising alternatives 1. Unlike conventional antibiotics, most AMPs are believed to exert their antimicrobial activity through membrane permeation and disruption, thus resistance is less likely to occur 2. AMPs with broad spectrum, unique mode of actions and rare resistant variants are increasingly coming into the focus as new treatment strategies for bacterial infections 3. However, there still exist many limitations of AMPs in efficient therapeutic application, such as high cost of synthesis, low stability and high toxicity 4. Various strategies have been employed in solving these problems, such as truncation of peptide, terminal modifications, peptidomimetics and so on 5. Hereinto, truncation of template peptide is widely used. Sometimes, shorter derivatives may be less toxic and highly active than the parent peptide, with a reduced cost of production correspondingly; for example a 15 residue shortened melittin 6, a shorter derivative of HP(2-20) 7 and a 17-mer peptide MSI-78(4-20) 8. Amino acid residue-selective substitution is a simple method for peptide modification. Maria Lnisa M *et al* improved peptide activity against G^+^ and fungus by replacing Gln^3^ with Pro^3^ in Temporin L 9.

**Figure 1:**
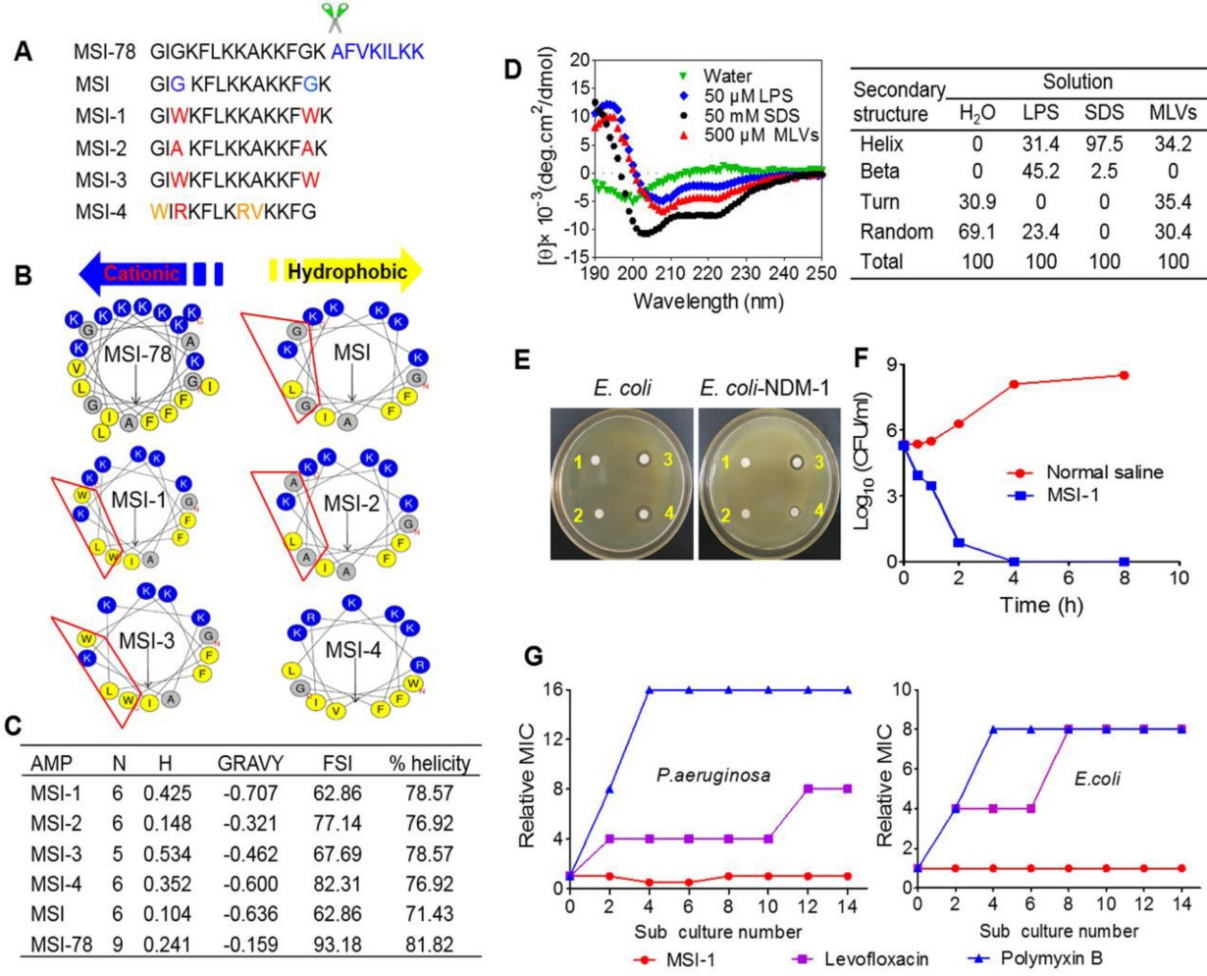
** Structure-activity relationships of peptides. (A)** The amino acid sequences of different peptide mutants. **(B)** Helical wheel projections of peptides. Amino acids in blue are positively charged, while in yellow are hydrophobic.** (C)** The key physicochemical properties of different peptide mutants. N: Net Charges; H: Hydrophobic; MW: Molecular Weight; GRAVY: the grand average of hydropathy; FSI: Fat-soluble Index. **(D)** CD spectra of MSI-1 in water, 50 μM LPS, 50 mM SDS and 500 μM MLVs (0.2 DPPG/DPPE +DPPG molar ratio system). **(E)** Disk diffusion antibacterial assay. 1: normal saline; 2: 10 µg penicillin G sodium salt; 3: 10 µg MSI-1; 4: 10 µg polymyxin. **(F)** Time-kill kinetics of MSI-1 (4× MIC) against *ndm-1*-carrying recombinant *E. coli* ('superbug'). **(G)** Drug resistance test for MSI-1.

Modifications performed on known peptides with excellent activity and low toxicity are more likely to be successful. MSI-78 (C_122_H_210_N_32_O_22_, MW: 2477.17), commercially known as pexiganan, is an analog of magainin-2 with 22-amino-acid and is developed for the treatment of infected diabetic foot ulcers 4, 10. However, it can lyse red blood cells, has poor biological stability, and is no more effective than already approved treatments for diabetic foot ulcers, which considerably diminish its effective applications 11. Therefore, in this study, we aimed to create a shorter and magainin 2-derived peptide analog that can maximize broad spectrum activity and had improved stability and safety. The analog peptides tested in this study were designed by truncating 14-amino-acids at the N-terminus of MSI-78 (Figure 1A). Herein, the truncated amino acid sequence, named MSI (C_75_H_129_N_21_O_14_, MW: 1549), was further modified to obtain other analog peptides. Considering the effect of the amino acid composition, cationic charges, amphiphilicity and α-helicity on the antimicrobial activity of AMPs, MSI-1 was designed by substituting Gly^3^ and Gly^13^, which are not conducive to the formation of alpha-helices, with the highly hydrophobic residue Trp to improve the degree of α-helix and amphiphilicity. Subsequently, to further confirm the importance of Trp on AMP activity, Gly^3^ and Gly^13^ were replaced by the poorly hydrophobic residue Ala to obtain the low amphiphilic peptide MSI-2. To clarify whether net charges and amphiphilicity significantly affected the activity and toxicity of the peptide, MSI-3 was designed by truncating the Lys residue at the C-terminus of MSI-1. Moreover, the cation-π interaction between Arg and Trp at the hydrophobic-hydrophilic interface was also considered in the design; thus, MSI-4 was obtained by substituting Gly^1^, Gly^3^, Lys^8^ and Ala^9^ with Trp^1^, Arg^3^, Arg^8^ and Val^9^.

In the present study, the antibacterial activities against drug-sensitive or -resistant G^+^/G^-^ bacteria, the selectivity of action against bacteria and the stability against violent physicochemical conditions of the modified analog peptides were evaluated to obtain a desirable peptide that improved activity with modulation of toxicity and stability. Then, we further detected its protective effects on acute systemic and/or local *E. coli*-infected mice and elucidated the possible mechanisms underlying its mode of action.

## Materials and Methods

### Peptides and reagents

All acetate of peptides (Purity: ≥ 98% (HPLC); Peptide Content (N%): ≥80.0%) were synthesized by GL Biochem Co., Ltd (Shanghai, China). Peptides are white fine amorphous powder and have easy to absorb moisture. They are very soluble in water, methanol, 0.1 M HCl and 0.1 M NaOH, and very slightly soluble in acetonitrile and ethanol. Mammalian cells, including HaCaT, GES-1, human L02, B16F10, A549 and H460, were obtained from Shanghai Institute of Cell Resource Center of Life Science (Shanghai, China). Polymyxin B, Penicillin G sodium salt, vancomycin, levofloxacin (Sangon Biotech Co., Ltd, Shanghai, China); LB nutrient broth, MHB and Columbia Blood Agar Base (Beijing SanYao Science & Technology Development Co., Beijing, China); LPS, propidium iodide, DAPI, pepsin, MTT, E-TOXATE kits and ONPG (Sigma-Aldrich, St. Louis, US); Anti-EGF and Anti-VEGF receptor 2 antibodies (Abcam, Cambridge, Britain). DPPG and DPPE (CordenPharma International, Plankstadt, Germany); BacteriaGen DNA Kit, PurePlasmid Mini Kit, HiFiScript 1st Strand cDNA Synthesis Kit and UltraSYBR Mixture (ComWin Biotech Co., Ltd, Jiangsu, China); TNF-α and IL-6 ELISA Kits (Shanghai Meixuan Biological Science and Technology Ltd, Shanghai, China); BCA Protein Assay Kit (Beyotime, Jiangsu, China). Other chemical reagents of analytical grade were purchased from commercial sources. The E-TOXATE kits were used to determine drugs. Test samples used in the study did not show detectable levels of endotoxin within the sensitivity limit of kit (0.1-1.0 EU/ml).

### Bacterial strains

Bacterial strains were purchased from the American Type Culture Collection (ATCC). All clinical strains were isolated from human clinical specimens by the Medical Laboratory Center of Zhongda Hospital (Nanjing, Jiangsu, China). *Ndm-*1, *tem-*1, *ctx-m-*1, *shv*-1 -carrying bacteria were constructed as previously described 12. The recombinant bacteria carrying drug-resistant genes were prepared in LB nutrient and induced by IPTG (1 mM) for 4 h before text. Other bacterial strains were respectively prepared in MHB and Columbia Blood Agar Base.

### Antimicrobial activity assay *in vitro*

#### Disk diffusion assay

Penicillin-resistant *E. coli* and *E. coli*-NDM-1 were evenly spread over Mueller-Hinton agar plates. The disks containing MSI-1, polymyxin, penicillin G sodium salt or PBS, were placed on the inoculated agar plates. The inhibition zone diameter of each disk was determined after 18 h incubation.

#### Broth microdilution assay

A standard micro-broth serial dilution assay was used to measure MIC of peptides. MIC was obtained by a microplate reader at 600 nm. MBC was determined as previously described 13.

#### Time-killing assay

Bacteria were incubated with MSI-1, penicillin G sodium salt, vancomycin or methicillin at 4× MIC. Samples were removed at 0, 0.5, 1, 2, 4, and 8 h for bacterial counts.

#### Resistance study

*P. aeruginosa* and *E. coli*, which were penicillin resistant but levofloxacin sensitive strains, were exposed to MSI-1, polymyxin and levofloxacin for MIC determination. Resistance study was performed as described previously 14. After fifteen similar serial passages exposing to the tested drugs (1/4× MIC), the relative MIC was identified as the ratio of MIC obtained from the given subculture to that of first-time exposure.

### The selectivity of action against bacteria

#### Hemolysis activity

Sheep red blood cells (sRBCs) were washed with saline for five times, re-suspended and then incubated with serially diluted peptides (final concentrations: 6.25, 12.5, 25, 50, 100, 200 and 400 μM) for 4 h. After centrifugation, OD_570_ of the supernatant was recorded. Cells incubated with melittin was detected for comparison. The hemolysis percentage was calculated as previously described 15.

#### Cytotoxicity towards eukaryotic cells

The cytotoxicity of peptides towards murine spleen cells (obtained from healthy murine with aseptic technique) and other eukaryotic cells (HaCaT, GES-1, human L02, B16F10, A549, H460) were determined by routine MTT method. Cells were incubated with serially diluted peptides (final concentrations: 1.563, 3.125, 6.25, 12.5, 25, 50, 100 and 200 μM) for 48 h_._

#### The binding ability of peptides to HaCaT cells and bacterial cell membranes

HaCaT cells was incubated with FITC-labeled peptide (8 μg/ml, MIC against bacteria) for 30 min. After washing, some cells were stained with DAPI and observed under an LSM 700 confocal laser scanning microscope (Carl Zeiss Meditec, Oberkochen, Germany); the other cells were subjected to flow cytometry. Meanwhile, *E. coli* were co-incubated with FITC-labeled peptide for 30 min and detected by flow cytometer (BD Biosciences, San Jose, CA, USA) and confocal microscopy.

#### Acute toxicity in mice

C57BL/6 mice (n=6/group) were intraperitoneally injected with MSI-1 (0, 10, 20, 40, 80 and 100 mg/kg/body weight, respectively). Animals were inspected for adverse effects for 30 min, and survival was monitored for 12 h thereafter. The toxicity severity was classified as Malik U *et al* previously described 16.

#### CD assay

MSI-1 were respectively dissolved in SDS (50 mM), LPS (50 μM), MLVs (500 μM, 0.2 DPPG/DPPE + DPPG molar ratio system prepared by the film-dispersion method) and ddH_2_O, with peptide final concentration of 0.1 mg/ml. CD values were measured at 37 °C with a spectrum of 190-250 nm by a CD spectrophotometer (MOS 450; quartz: 0.1 cm; bandwidth: 1 nm).

### Stability analysis

#### pH stability

MSI-1 at 2 mg/ml in PBS (pH 7.0), aqueous HCl (pH 3.0) and aqueous NaOH (pH 11.0) were incubated for 2, 4, 12 and 24 h at 37 °C. The remaining MSI-1 was measured by HPLC. In addition, the bacteria colonies survived 1× MIC of HCl or NaOH treated peptide were recorded simultaneously. That is, after 18 h coincubation, the absorbance which represents the bacteria colonies survived, was recorded by a microplate reader at 600 nm 17.

#### Protease stability

MSI-1 and protease solution (pepsin or trypsin) were prepared in 0.1 M NH_4_HCO_3_ buffer (pH 8.2) to final molar ratio of 300:1 and incubated at room temperature. Aliquots of the mixture were extracted periodically and diluted with water/acetonitrile (3:2, v:v) containing 2% TFA for HPLC detection or for antibacterial activity assay 18.

#### Thermal stability

MSI-1 were placed at 37 °C for 7 days, repeated multigelation at -80 °C for 10 times or boiled at 100 °C for 1 h, and then subjected to HPLC analysis or antibacterial activity evaluation as above.

#### Plasma stability

MSI-1 stability in plasma or serum was evaluated using an established method 19. Briefly, MSI-1 solution (2 mg/ml) was mixed with fresh mice plasma or serum (4:1, v:v) at 37 °C. At indicated time points, samples were denatured with equal volumes of 6 M urea and subjected to HPLC analysis or antibacterial activity evaluation.

#### Salt stability

*E. coli* were incubated with different concentrations of MSI-1 supplemented with NaCl (50, 100, 200 and 400 mM) for 18 h. The salt stability of MSI-1 was assessed by the mean MIC at per salt concentration.

### Antimicrobial activity assay *in vivo*

Manipulations of animals were performed in accordance with the Guideline for the Care and Use of Laboratory Animals published by the US National Institutes of Health (NIH Publication No. 85-23, revised 1996), and was approved by the Experimental Animal Ethic Committee of China Pharmaceutical University and the Science and Technology Department of Jiangsu Province (SYXK 2016-0011).

#### The protective effects of MSI-1 on acute systemic *E. coli* infection in mice

BALB/c mice (5-6 weeks) were purchased from the Laboratory Animal Center of Yangzhou University (Yangzhou, Jiangsu, China) and intraperitoneally (ip) injected with 500 μl (5× 10^8^ CFU/ml) of penicillin resistant-*E. coli* isolates. Mice in MSI-1 treated groups (10, 5 and 2.5 mg/kg, n=16/group) and polymyxin treated group (5 mg/kg) were administered after 2 h post bacterial inoculation. After 12 h, six mice in each group were anesthetized with pentobarbital sodium (50 mg/kg body weight, i.p) and euthanized by cervical dislocation to count bacterial colonies in blood, lung, spleen, kidney and liver. Lung homogenates were collected for Gram staining. Moreover, serum TNF-α and IL-6 were measured by ELISA kits. The procedure of H&E staining for mouse lung was adapted from a published report 20. The survival and weight of other mice were monitored daily up to 7 days.

#### The protective effects of MSI-1 on local *E. coli* infection in scalded mouse model

To further investigate whether MSI-1 could protect from bacterial infection of the skin, mice were anesthetized with pentobarbital sodium (50 mg/kg, i.p) and shaved on the dorsal surfaces. The depilated skin was secured with 2-cm-diameter plastic containers that filled up with boiling water at contact time 10 s. Afterwards, a full-thickness wound (2 cm in diameter) was cut on the burned skin of each mouse. Penicillin-resistant *E. coli* suspension (100 µl, 1× 10^10^ CFU/ml) was slowly inoculated over wound with a pipette tip, and a gentle stream of air was aimed over the inoculation site until skin appeared wet but absent of any standing volume from the inoculum suspension. Two hours later, vehicle (3% CMC-Na), MSI-1 or polymyxin, which was suspended in sterile CMC-Na (3%) in PBS at concentration of 10, 5, 2.5 mg/ml (MSI-1) or 5 mg/ml (polymyxin), was inoculated on the same area with a pipette tip, 1 ml/kg, once a day (n=15 per group). Mice were fed in cages individually to avoid cross infection and were treated daily for 7 consecutive days. At 3^rd^ and 7^th^ days, cutaneous wound healing and bacteria counts in skin lesion were measured. Moreover, 12 h after the last treatment, blood samples were collected from the retro-orbital venous plexus under anesthesia (pentobarbital sodium, 50 mg/kg). Finally, all animals were euthanized by cervical dislocation to collect the lung samples for counting live bacteria. The contents of TNF-α and IL-6 in skin homogenates and serum were measured by ELISA. Skin lesion was fixed by 4% paraformaldehyde for H&E staining. The expression levels of EGF and VEGF in skin lesion were examined by immunohistochemistry. In addition, the wound scratch assay was also performed on HaCaT cells to further test whether MSI-1 improved wound healing by promoting keratinocyte migration.

### The potential antibacterial mechanisms *via* bacterial membrane-disruption effect

#### Zeta potential detection

Bacteria were mixed with MSI-1 (2, 8, 32 μg/ml). After 30 min incubation, the zeta potential was measured with a ZetaPlus particle size analyser (Brookhaven Instruments Corporation, Holtsville, NY) 21.

#### Flow cytometry assay

Bacterial suspension was added with FITC-labeled MSI-1 (32 μg/ml), incubated at 37 °C for indicated time and ultimately detected by flow cytometer after washing off the unbound peptide.

#### LPS binding assay

To study the effect generated by interactions between LPS and MSI-1, we monitored the antimicrobial activity of MSI-1 at 1/2×, 1× and 2× MIC against *E. coli*, in the presence of increasing LPS concentrations. *E. coli* suspension (100 μl/well) was added with isovolumetric LPS-MSI-1 mixtures. After 16 h incubation, bacteria growth was measured at 600 nm. Additionally, the non-fluorescent partner LPS, ranging from 2000 to 0.98 μg/ml with 2-fold dilution, was titrated against a fixed concentration of the fluorescent partner (FITC-labeled MSI-1, 4× MIC) for indicated time. That is, we performed Microscale Thermophoresis (MST) experiment to analyze the interaction between MSI-1 and LPS by calculating the apparent Kd as Seidel SA *et al* reported using a Monolith NT.115 instrument (Nanotemper Technologies, Munich, Germany) 22, 23.

#### Detections for membrane integrity of bacteria

*E. coli* were mixed with different concentrations of MSI-1, incubated for 2 h, resuspended by PI solution (20 μg/ml), and ultimately subjected to flow cytometry for PI uptake assay. Calcein-loaded liposomes were prepared to imitate bacterial membrane. Serially diluted peptide was added into liposomes (1:1, v:v) after obtaining the fluorescence intensity at λ_ex_/λ_em_=490/520 nm. Once MSI-1 addition, fluorescence intensity was detected at 0, 3, 5, 10, 15 and 20 min. Calcein release rate was calculated as previously described 24. To observe bacterial morphology, *E. coli* treated with MSI-1 (32 μg/ml) or PBS for 2 h was collected, fixed by 2.5% glutaraldehyde and subjected to scanning electron microscopy (SEM) assay (SUI510, Hitachi, Japan)*.* In addition, *E. coli* were resuspended in reaction buffer (60 mM Na_2_HPO_4_^.^ 7H_2_O, 40 mM NaH_2_PO_4_^.^ H_2_O, 10 mM KCl, 1 mM MgSO_4_.7H_2_O, 0.27% 2-Mercaptoethanol), incubated with ONPG (2.5 mg/ml) and MSI-1 for 2 h. Bacteria envelope integrity was calculated by the percentage of O-nitrophenol production.

### The potential antibacterial mechanisms *via* bacterial DNA-binding effect

#### Bacteria- growth curve and viability

*E. coli* were mixed with sub-MICs MSI-1 and OD_600_ value was recorded every 2 h throughout 26 h of coincubation. In addition, bacteria were suspended with nutrient broth containing MSI-1 and subjected to routine MTT assay after 24 h incubation.

#### DNA binding assay

Bacterial genomic DNA was obtained by a BacteriaGen DNA Kit and quantified by a Thermo Scientific NanoDrop™ 2000c Spectrophotometer. Bacterial genomic DNA samples were incubated with isovolumetric MSI-1 at room temperature for 15 min. The gel retardation assay for DNA shift was performed by 1% w/v agarose gel (pre-stained by SYBR Green I) electrophoresis. In UV spectroscopic study, different concentrations of peptide were titrated into genomic DNA (0.5 μg/ml). After 15 min to equilibrate, the UV spectra for DNA and MSI-1-DNA complex were recorded at wavelength range of 230-350 nm. In addition, DNA dissolved in Tris-HCl (pH 7.4, C_DNA_=0.1 mg/ml) was mixed with EB solution (5 μM). After 10 min incubation in the dark, the EB-DNA mixture was added with different concentrations of MSI-1. Thirty minutes later, the fluorescence spectra were recorded at excitation wavelength 535 nm 25 to measure the competitive binding of MSI-1 and EB with bacterial DNA. CD spectra of DNA or DNA-peptide complexes (C_DNA_=0.25 mg/ml, C_MSI-1_=1/2 or 2× MIC) were measured with a spectrum of 220-350 nm by a CD spectrophotometer. Secondary structure change of *E. coli* DNA was analyzed 25. Moreover, the intracellular changes in* E. coli* morphology was observed by a JEOL 1230 transmission electron microscopy (TEM) after 2 h coincubation with MSI-1.

### The potential antibacterial activity *via* affecting the biological functions of DNA and protein

#### *In vivo* replication assay for β lactamases encoding genes

Exponentially growing cells, including NDM-1 (NewDelhi Metallo)-β-lactamase produced multidrug resistant bacteria and *tem-1*, *ctx-m1*, *shv-1* type β-lactamases produced drug-resistant bacteria, were treated with peptide for 2 h. After centrifugation, the pellets were collected for RNA extraction and subsequent PCR reaction as previously described to detect the expression levels of genes 26, 27. After amplification, samples were subjected to agarose gel assay and visualized under UV light.

#### The interaction of DNA synthesis-related genes with MSI-1

Gel electrophoresis process was used to detect the interaction of *E. coli* DNA synthesis-related genes with MSI-1 after 15 min coincubation. The primers used for PCR were listed in Table 3.

#### The detection of bacterial protein production

Bacteria treated with MSI-1 for 8 h were sonicated to obtain bacterial protein. SDS-PAGE and BCA assay were performed to detect the expression of total bacterial proteins and the total protein concentrations of bacteria.

### Statistical analysis

All data were performed by using SPSS 22.0 software and presented as the mean ± SD. Comparison among groups was made by one-way ANOVA. The significance of the differences among means was evaluated using the two-tailed paired Student *T* test (^*^p < 0.05, ^**^p < 0.01).

## Results

### The characterizations of designed peptides

The amino acid sequences, α-helical wheel and predicted physiological properties of the analog peptides exhibited typical cationic, amphiphilic and α-helical properties. MSI-1 (C_93_H_143_N_23_O_14_, MW: 1807.3), with the Trp^3^ and Trp^13^ replacements, achieved a significant increase in amphiphilicity compared with MSI. However, despite its higher FSI, the amphiphilicity of MSI-2 (C_77_H_133_N_21_O_14_, MW: 1577.1) was lower than that of MSI-1 due to the presence of Ala, with a hydrophobicity of 0.148, hydrophobic moment of 0.637 and GRAVY of -0.321, indicating that Trp was very important for peptide amphiphilicity. After the Lys residue at the C-terminus of MSI-1 was truncated, MSI-3 (C_87_H_131_N_21_O_13_, MW: 1679.2) exhibited decreased net charges (+5) and slightly higher hydrophobicity with a hydrophobicity of 0.534, hydrophobic moment of 0.718 and GRAVY of -0.462. For MSI-4 (C_84_H_137_N_25_O_13_, MW: 1705.2), there was a cation-π interaction between Arg and Trp at the hydrophobic-hydrophilic interface, which resulted in enhanced FSI and hydrophobic moment, but its percent helicity was low (Figure 1A-C).

### Potent antibacterial activity of peptide mutants *in vitro*

As summarized in Table 1, MSI-1 and MSI-3 displayed superior antimicrobial activity against both G^-^ and G^+^ bacteria compared to the parent peptide with an MIC of 4-16 μg/ml. MSI-4 showed reduced activity with an MIC of 16-64 μg/ml. Interestingly, MSI-1 and MSI-3 exhibited more than two times greater activity than MSI-78 when examining drug-resistant bacteria, including penicillin- or methicillin-resistant bacteria. Furthermore, in the disk diffusion test, MSI-1 displayed a diameter of inhibition zone ˃20 mm against penicillin-resistant pathogens (Figure 1E). In addition, as indicated in Table 2, MSI-1 displayed a potent bactericidal effect on drug-resistant bacteria, which was comparable to that of melittin. Interestingly, the time-kill curves showed that MSI-1 (4× MIC) exerted a potent bactericidal effect on *ndm-1*-carrying recombinant *E. coli*, thoroughly eliminating the bacteria by 4 h after coincubation (Figure 1F). Moreover, as shown in Figure 1G, after 15 subcultures following the initial exposure, the relative MIC of MSI-1 against *E. coli* and* P. aeruginosa* remained stable, reflecting no emergence of resistant bacteria. Thus, MSI-1 has a strong antibacterial activity, broad antibacterial spectrum, and low potential for emergence of resistance.

### Rapid bactericidal efficiency of MSI-1 against clinically isolated strains *in vitro*

**Figure 2:**
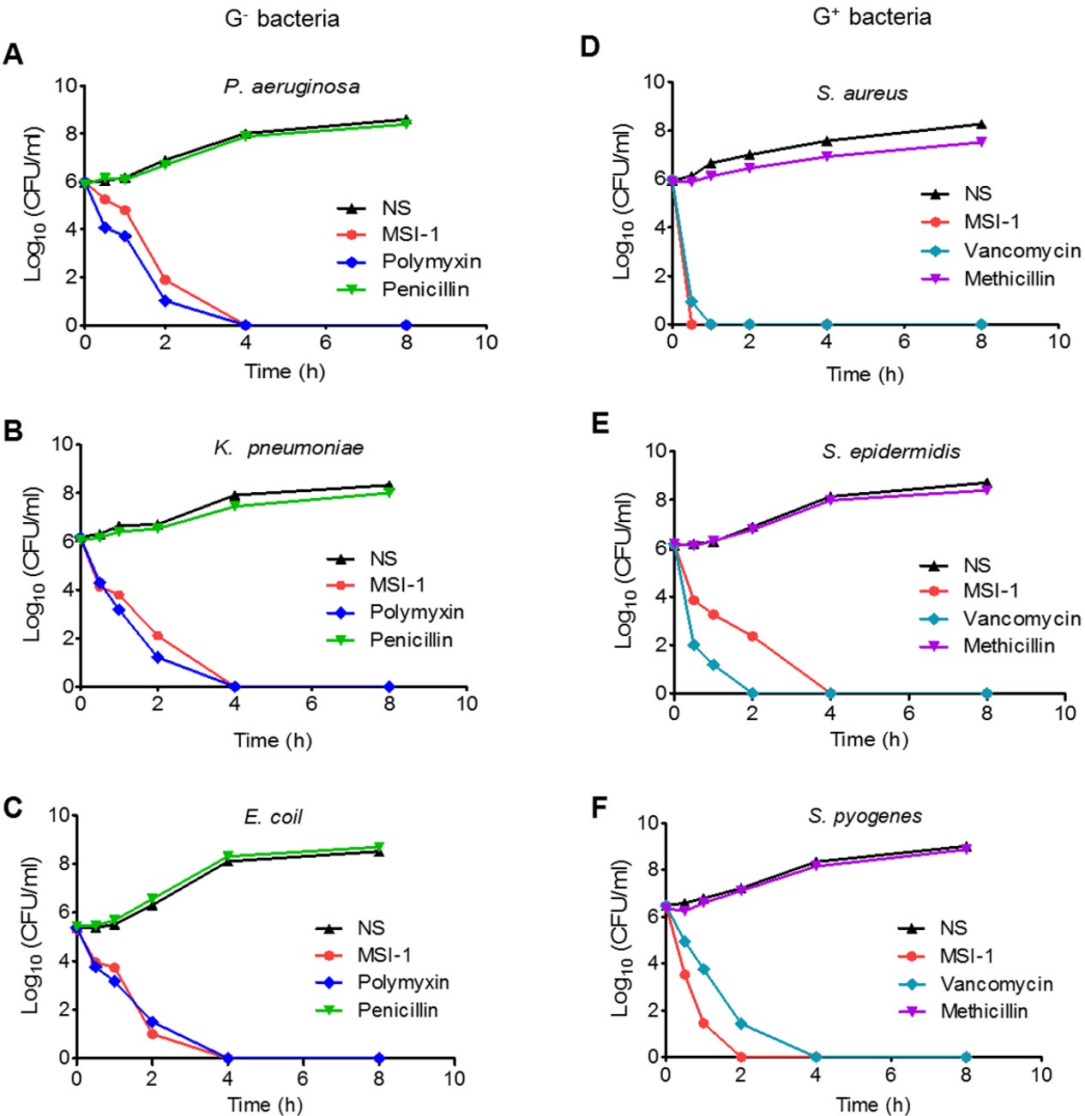
The time-kill curves of MSI-1 against penicillin-resistant G^-^ bacteria and methicillin-resistant G^+^ bacteria. The penicillin-resistant G^-^ bacteria include (A) *P. aeruginosa*, (B) *K. pneumonia* and (C) *E. coli*; the methicillin-resistant G^+^ bacteria include (D) *S. aureus*, (E) *S. epidermidis* and (F) *S. pyogenes*. NS: Normal saline.

As Figure 2 shows, MSI-1 exerted rapid killing effects on *S. epidermidis*, with a reduction in CFU counts of approximately 5 log units and thorough bacteria elimination within 240 min. The same trend was observed in *P. aeruginosa, E. coli* and *K. pneumoniae*. These observations suggest that MSI-1 has a rapid bactericidal effect against both penicillin-resistant G^-^ bacteria and methicillin-resistant G^+^ bacteria.

### A desirable selectivity of action of MSI-1 against bacteria

**Figure 3:**
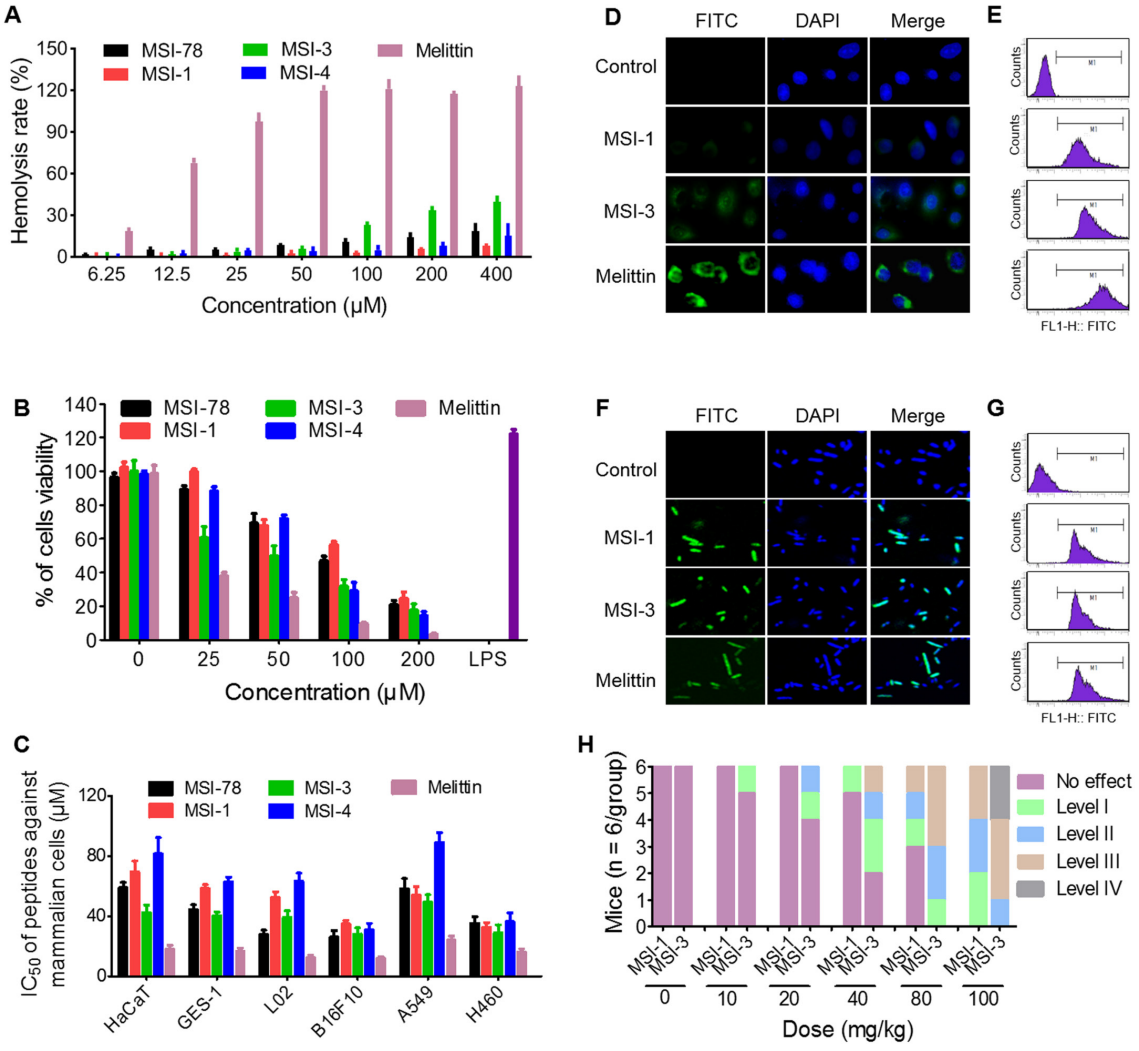
** The selectivity of MSI-1 against bacteria. (A)** Hemolytic activity toward SRBCs. 0.1% Triton X-100 or PBS was used as positive (100% hemolysis) or negative (0% hemolysis) controls. MTT assay for the cytotoxicity of the modified analog peptides (final concentrations: 1.563, 3.125, 6.25, 12.5, 25, 50, 100 and 200 μM) toward murine spleen cells **(B)** and other eukaryotic cells **(C)**. LPS, which has a strong effect on promoting proliferation of mouse spleen cells, was used as positive control. IC_50_: half maximal inhibitory concentration. **(D-G)** The binding ability of peptides (8 μg/ml, MIC against bacteria) to HaCaT cells and bacterial cell membranes. Confocal laser scanning microscope (left) or flow cytometry (right). Green: FITC-labeled peptide. Blue: DAPI for nucleic acid staining. **(H)** Acute toxicity of peptide intraperitoneal injection. The toxicity severity: No effect; Level-1, narrowing of the eyes; Level-2, crouching and cuddling; Level-3, convulsions and mania; Level-4: death.

The hemolysis activity and cytotoxicity of peptides to mammalian are important problems that hinder the application of peptides. Data from hemolysis test demonstrated that three mutants with potent antibacterial activity exhibited low hemolysis activity (HC_50_ > 400 μM) against to sRBCs (Figure 3A), compared with melittin which has a rapid cytolytic action and destabilizes membranes of different cell types 28. Interestingly, at peptide doses up to 400 μM, the hemolysis rate was higher than 30% in the MSI-3 group but lower than 10% in the MSI-1 group.

Furthermore, the cytotoxicity studies indicated that the effects of MSI-1 and MSI-4 on the survival of mouse spleen cells were similar to that of the parent peptide, while the toxicity of MSI-3 was significantly higher (Figure 3B). Moreover, the effective antimicrobial concentration of MSI-1 *in vitro* (4.4-8.8 μM) was far less than its IC_50_ (50% inhibitory concentration) value on the eukaryotic cells (Figure 3C). To further investigate the selectivity of MSI-1, the fluorescence intensity of FITC-MSI-1 on cells and bacteria was detected after 30 min of coincubation with HaCaT cells or penicillin-resistant *E. coli*. As shown in Figure 3D, a small amount of MSI-1 was associated with the surface of HaCaT cells, which was less than that of MSI-3. However, both MSI-1 and MSI-3 showed weaker adhesive capacity than melittin. Notably, these three peptides all displayed a powerful binding ability to bacteria as reflected by the strong fluorescence intensity observed with *E. coli* (Figure 3F). Similarly, the data from flow cytometry also manifested that the efficiency of MSI-1 adhesion to HaCaT was slighter than that of MSI-3. However, both mutations possessed high binding affinity to bacterial membrane (Figure 3E and 3G). These results corresponded with the data from the hemolysis and cytotoxicity assays, suggesting that MSI-1 has a high selectivity for bacteria. *In vivo*, no immediate adverse events were noted for MSI-1 at a dose of 20 mg/kg. At approximately 5 min post injection with a dose of peptide above 40 mg/kg, one mouse in the MS-1-treated group exhibited narrowing of the eyes. However, two mice in the MSI-3-treated group displayed narrowing of the eyes, one mouse was crouching and cuddling, and the other mouse showed convulsions. No deaths were observed for any of the treated mice when the peptide dose was no more than 80 mg/kg and when the mice recovered 2 h after treatment. Importantly, some of the mice in the MSI-3-treated group died as the peptide dose reached 100 mg/kg, indicating that MSI-3 not only had potent antibacterial activity but also exhibited a higher acute toxicity in mice than MSI-1 (Figure 3H).

### Desirable stability of MSI-1 against violent pH, high NaCl concentration and temperature variations

**Figure 4:**
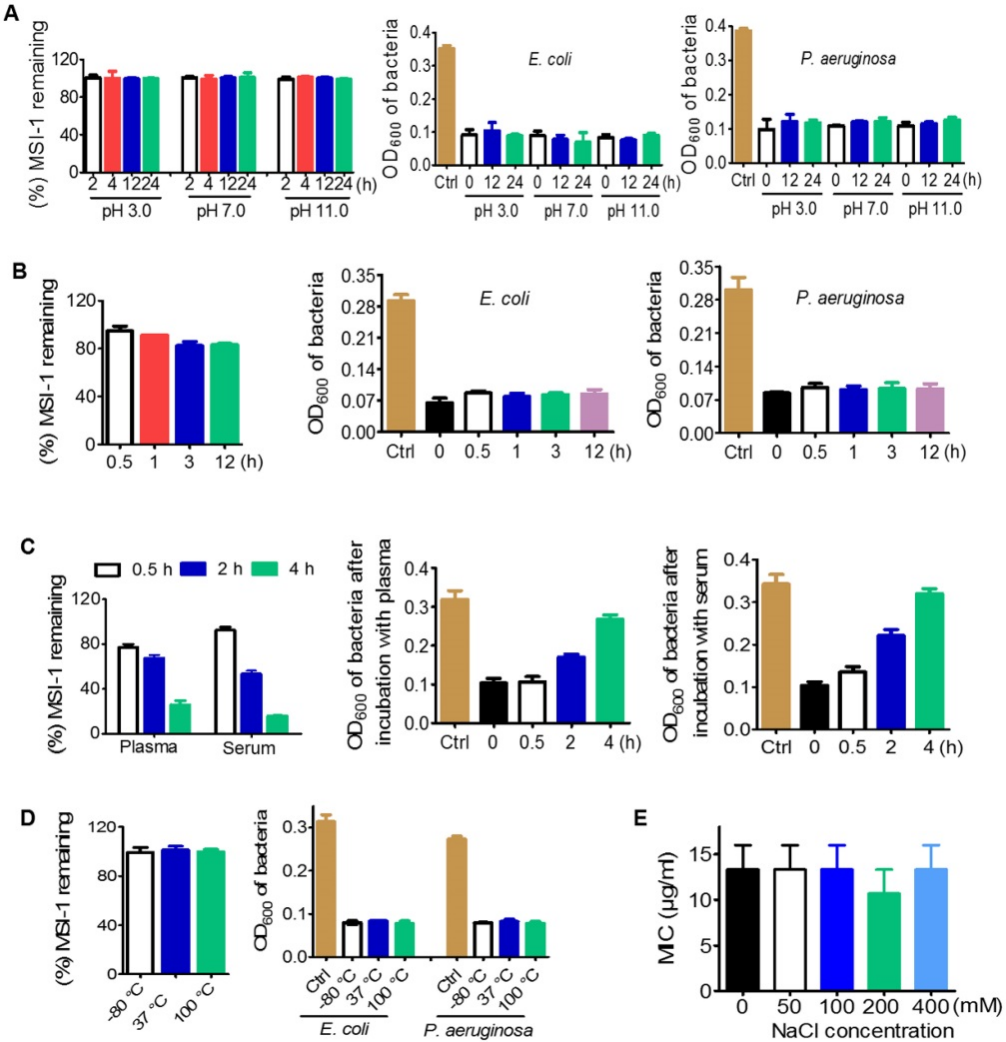
** Stability of MSI-1 against pH, protease, thermal, plasma and high salt. (A)** pH stability in PBS (pH 7.0), aqueous HCl (pH 3.0) and aqueous NaOH (pH 11.0). **(B)** Protease stability in pepsin or trypsin (final molar ratio of MSI-1 *vs* protease of 300:1). **(C)** Plasma or serum stability in 20% fresh mouse plasma or serum. **(D)** Thermal stability after incubation at 37 °C for 7 days, repeated multigelation at -80 °C for 10 times or boiling at 100 °C for 1 h. Left: remaining MSI-1 measured by HPLC; right: turbidimetry assay for peptide bacteriostatic activity against penicillin-resistant *E. coli* and *P. aeruginosa* after treatment with acid, base, protease, boil and plasma/serum. **(E)** Salt stability at different salt concentrations.

As determined by HPLC, the presence of HCl or NaOH had no effect on the peptides with increasing incubation time. The percentage of remaining MSI-1 could be maintained at 99%-100% within 24 h (Figure 4A). Similarly, the treatment of HCl (pH 3.0) and NaOH (pH 11.0) did not affect the MIC of MSI-1 against bacteria. Moreover, in pepsin solution, the peak time of MSI-1 changed from 6.187 min at 0 h to 6.792 min at 0.5 h or to 6.796 min at 12 h, indicating that some peptide hydrolysates might be produced. Interestingly, the peptide hydrolysates also exhibited antibacterial activities (Figure 4B). However, MSI-1 had poor stability in trypsin, and the amount of remaining peptide was less than 10% after 0.5 h of coincubation (data not provided). After incubating with 20% fresh mouse plasma, MSI-1 was gradually degraded over time, and the remaining peptide was approximately 77.0% at 0.5 h and 67.5% at 2 h, and the antibacterial activities gradually decreased to 97.2% (0.5 h) and 60.7% (2 h). Similarly, the incubation with serum also led to MSI-1 degradation, and the remaining peptide was 92.5% (0.5 h) and 53.5% (2 h) while the inhibition rate against bacteria was 76.8% and 46.9%, respectively (Figure 4C)**.** Meanwhile, we found that MSI-1 maintained its structure and potent antimicrobial activity after incubation at 37 °C for up to 7 days, repeated multigelation at -80 °C for 10 times or boiling at 100 °C for 1 h (Figure 4D). For some AMPs, their antibacterial activities are greatly attenuated by high NaCl concentrations 29. As shown in Figure 4E, when various concentrations of NaCl were supplemented, the antimicrobial activity of the peptide was not affected, even when the NaCl concentration increased from 100 to 400 mM, which was much higher than human physiological salt concentration of 150 mM; however, the mean MIC still stayed at 13.3 μg/ml, suggesting the high salt-resistance of MSI-1. Overall, MSI-1 has a strong resistance against drastic pH changes, temperature variations, pepsin and high NaCl concentrations.

### Effective antimicrobial activity of MSI-1 *in vivo*

#### MSI-1 attenuated acute systemic *E. coli* infection in mice

**Figure 5:**
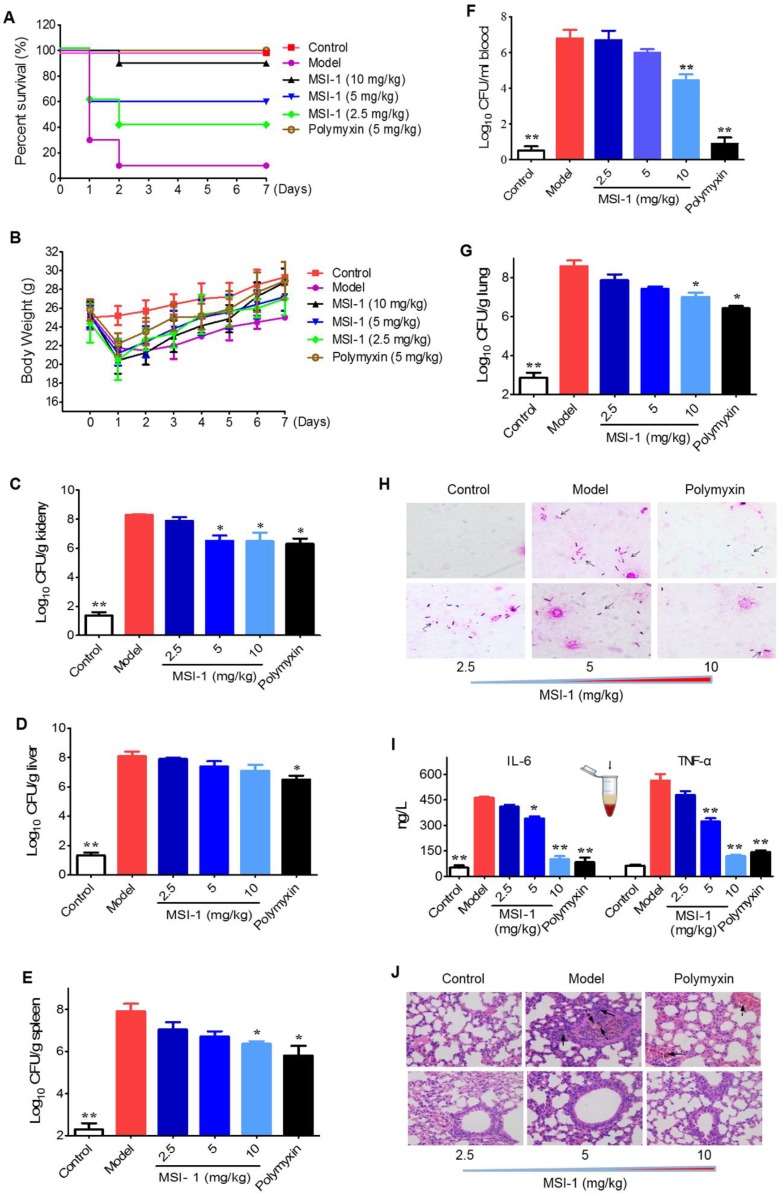
** The effects of MSI-1 on acute systemic *E. coli* infection in mice. (A-B)** The survival and body weight of mice during the observation period of 7 consecutive days. **(C-G)** Bacterial colonies in mice blood, lung, kidney, liver and spleen samples.** (H)** Gram staining for mouse lung homogenates.** (I)** Serum TNF-α and IL-6 contents. **(J)** H&E staining for mouse lung. ^*^ P<0.05 and ^**^p<0.01 *vs* the model group.

The mouse survival rates in the MSI-1-treated group were enhanced by 80%, 50% and 30% when compared with those in the model group (Figure 5A). Moreover, both MSI-1 and polymyxin exhibited a marked weight maintenance effect to some extent (Figure 5B). MSI-1 administration could significantly reduce bacterial colonies, especially at doses up to 10 mg/kg, where an approximately 2 log unit reduction in CFU could be observed (Figure 5C-G). Gram staining for lung homogenates more intuitively demonstrated that the peptide could reduce the total viable bacteria in lung (Figure 4H), suggesting that the improved mice survival was associated with decreased bacterial titers in blood or organs. Elevated serum IL-6 and TNF-α are capable of predicting the severity of bacterial infection 30. Here, the secretion of these two proinflammatory factors in the model group was significantly increased 12 h post-infection. However, followed by the elimination of bacteria in the mouse body, MSI-1 also obviously decreased IL-6 and TNF-α levels (p<0.05, Figure 5I), especially in the 10 mg/kg MSI-1 treated group, which displayed decreased rates of approximately 70%~80%. Actually, similar findings were obtained by Lee *et al* for HPA3P2, a helix-PXXP-helix peptide 31 and by Ma *et al* for Cbf-14, a helix-RLLR-helix peptide 24. The uncontrolled overproduction of proinflammatory cytokines, such as TNF-α, IL-1β and IL-6, induced by microbial pathogens may lead to diffuse alveolar damage with intrapulmonary hemorrhage, edema, tissue necrosis and lung damage 32. Here, *E. coli* infection resulted in severe diffuse alveolar damage with intrapulmonary hemorrhage and edema accompanied by substantial inflammatory cell infiltration, as evidenced by H&E staining (Figure 5J). MSI-1 administration significantly alleviated lung lesion, indicating that MIS-1 could reverse lung injury caused by penicillin-resistant* E. coli*, indicating that MSI-1 attenuates acute systemic *E. coli* infection in mice.

#### MSI-1 improved local *E. coli* infection in a scalded mouse model

**Figure 6:**
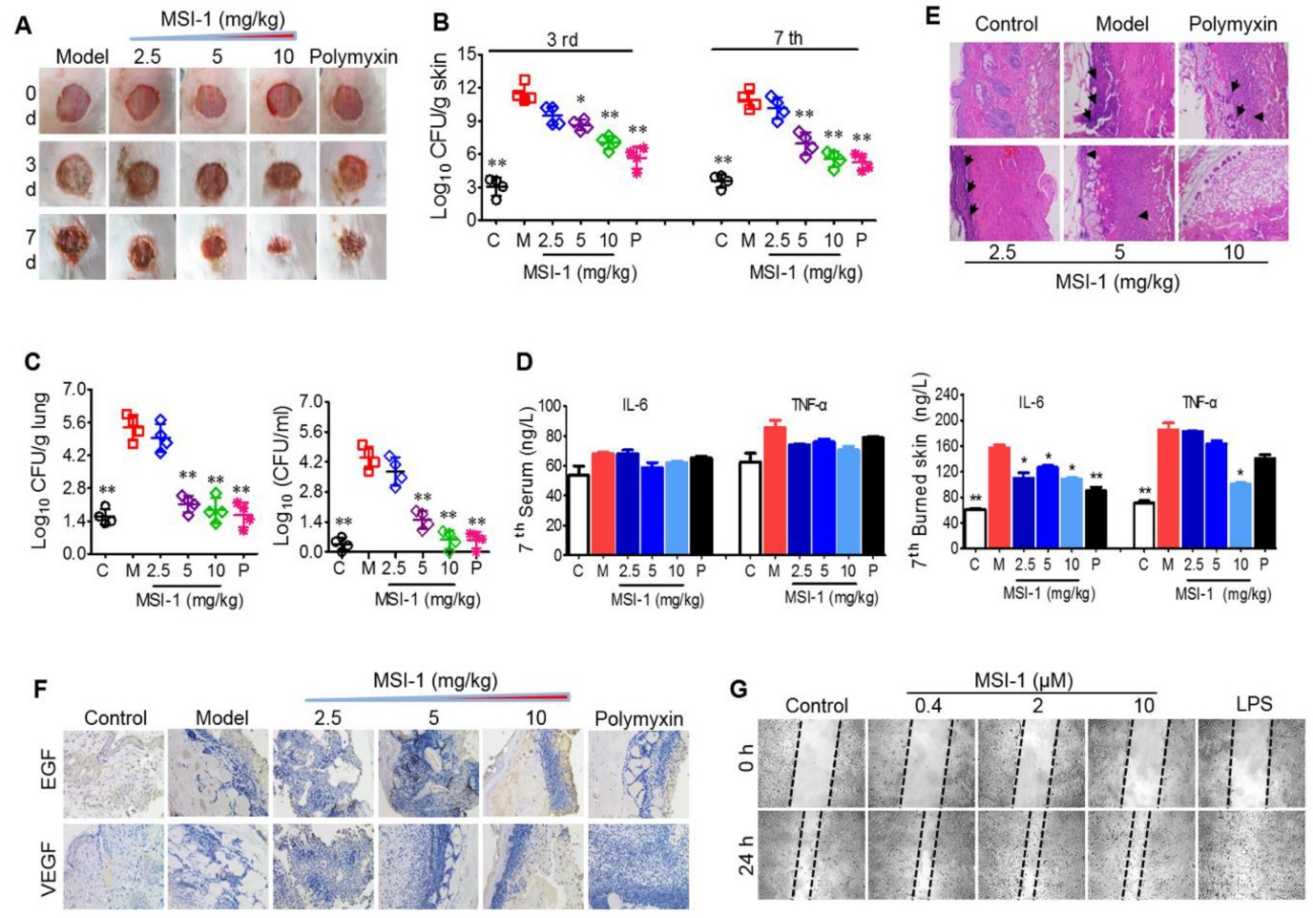
** The effects of MSI-1 on local *E. coli* infection in a scalded mouse model. (A)** Photographs of wounds treated with MSI-1 for 0, 3 and 7 days (scale bar=2 mm). **(B)** The subeschar bacterial colonies at 3^rd^ and 7^th^ days. **(C)** Bacterial colonies in mouse lung and blood at 7^th^ day.** (D)** TNF-α and IL-6 contents in mouse serum and skin lesion homogenate at 7^th^ day. **(E)** H&E staining for the histological change of mouse skin at 7^th^ day. Black arrows indicate inflammatory cell infiltration (scale bar=50 μm). **(F)** Immunohistochemistry for the expression of EGF and VEGF in skin. **(G)** Cell scratch assay for cells migration. LPS, which has a potent promoting migration effect on HaCaT cells, is served as the positive control. ^*^ P<0.05, ^**^ p<0.01 *vs* the model group.

Next, the effects of MSI-1 on an *E. coli*-infected scalded mouse model were studied in 7-day-long assays. In the presence of bacterial infection, mouse wound healing was slow without drug treatment, showing nonreduced unclosed wound areas on the 3^rd^ and 7^th^ days and purulent wounds on the 7^th^ day. However, MSI-1 significantly improved wound closure and inhibited purulence secretion from the infected wounds, especially MSI-1 at a dose of 10 mg/kg (Figure 6A). Compared with normal mice, *E. coli*-infected mice showed significantly higher subeschar bacterial counts. The number of colonies in peptide-treated mice was decreased in a dose-dependent manner. At a dose of 10 mg/kg, subeschar colonies were reduced to 38.6% after 3 days and to 49% after 7 days (Figure 6B). On the 7^th^ day (treatment was over), bacterial colonies in the lung and blood were obviously decreased in the MSI-1 (5 and 10 mg/kg) and polymyxin B groups (p<0.01 *vs* the model group, Figure 6C). Because inflammation plays an important role in wound healing, we also detected the production of TNF-α and IL-6 and found that serum TNF-α and IL-6 had no significant difference between control and bacteria-infected mice after 7 days of treatment, while these two cytokines in skin lesion homogenate were strongly reduced by MSI-1 (10 mg/kg) (see Figure 6D). H&E staining showed that the granulation tissues were much thicker in the MSI-1 (10 mg/kg) group. The congestion and inflammatory cell infiltration could also be attenuated by the peptide (Figure 6E), suggesting that MSI-1 may decrease the systemic inflammatory response induced by local infection by inhibiting the bacterium from spreading systemically. To clarify whether MSI-1 improved wound closure by regulating EGF and VEGF, we examined the expression levels of EGF and VEGF in the burned and infected skin and confirmed that MSI-1 had no obvious effect on the expression of EGF and VEGF (Figure 6F). In addition, a cell scratch assay also demonstrated that MSI-1 could not promote the migration of HaCaT cells (Figure 6G). The data above indicate that MSI-1 significantly improve wound closure in the *E. coli*-infected burn-wound model.

### MSI-1 damaged the membrane integrity of penicillin-resistant bacteria

**Figure 7:**
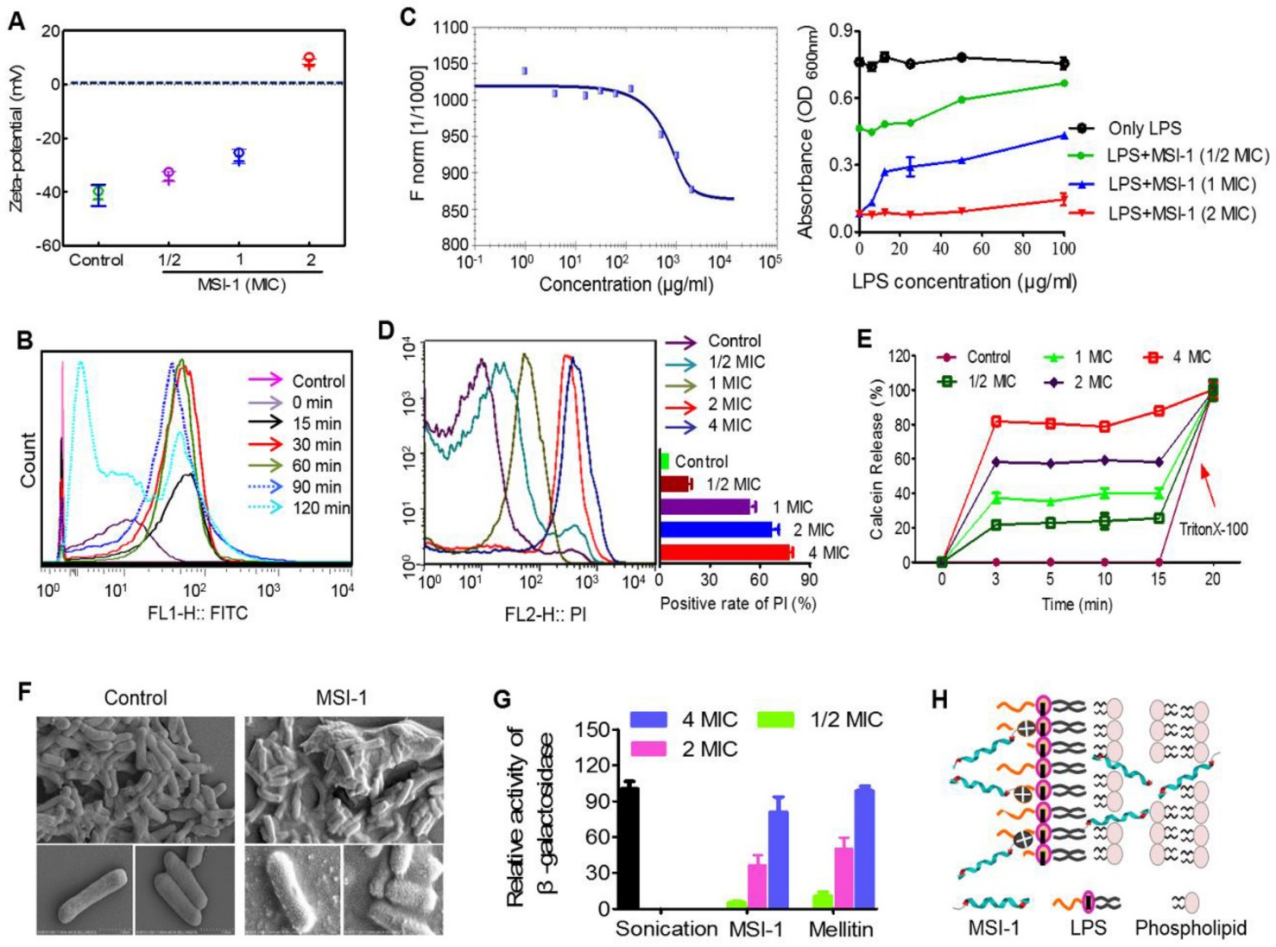
** The potential antibacterial mechanisms on the bacterial membrane. (A)** The zeta-potential detection for* E. coli* treated with peptide.** (B)** Flow cytometry for MSI-1 affinity to the bacterial membrane after 0, 15, 30, 60, 90 and 120 min of coincubation. **(C)** LPS binding assay. Left: MST assay for the interaction between FITC-MSI-1 and LPS. Binding data were plotted using the Hill equation. Right: In the presence of increasing LPS concentrations, the antibacterial activities of MSI-1 at 1/2, 1, and 2× MIC against penicillin-resistant *E. coli*. **(D)** PI uptake assay for membrane integrity of bacteria. A histogram quantified the proportion of PI-positive stained cells. **(E)** The release rates of Calcein from MSI-1-treated liposomes at indicated time. 0.1% TritonX-100 was set as positive control.** (F)** SEM of *E. coli* after MSI-1 (4× MIC) treatment for 1 h. Magnification=25,000× or 50,000×. **(G)** β-Lactamase activity in the culture media of *E. coli*. Herein, activities of the MSI-1-treated groups were shown relative to that of the sonicated bacteria, whose activity was defined as 100%. **(H)** The membrane rupture properties of MSI-1 against G^-^ bacteria.

*E. coli* surface was negatively charged (~41 mV), while MSI-1 could neutralize the negative charges in a dose-dependent manner (from -34.19 mV to 8.59 mV, Figure 7A). Moreover, MSI-1 aggregated on the bacterial surface after charge neutralization. Because a large number of FITC-labeled MSI-1 was detected on the bacterial surface, the fluorescence intensity gradually increased as the coincubation time increased. However, after 120 min of incubation, the fluorescence intensity declined instead of increased, which may be due to the insertion of peptide into bacterial cells or the destruction of the bacterial cell membrane (Figure 7B). As LPS is a major component of the *E. coli* membrane and contributes to the negative charges on the bacterial surface, we detected the binding ability of FITC-MSI-1 to pure LPS. The results from the MST assay showed that the apparent Kd was 100±5.77 μg/ml (Figure 7C, left), suggesting a binding reaction between MSI-1 and LPS. Subsequently, we further investigated the effect generated by the interaction of LPS and MSI-1 and found that there was a clear negative effect of LPS on the antimicrobial activity of MSI-1, beginning at LPS concentrations of 12.5 µg/ml (1× MIC peptide) and 100 µg/ml (2× MIC peptide), and this effect became more pronounced as the LPS concentration increased (Figure 7C, right).

In the PI uptake test, the PI-positive bacteria increased with increasing peptide concentration, and the positive rate reached 80.4% at 4× MIC (Figure 7D). Furthermore, MSI-1 treatment caused rapid membrane rupture, inducing over 80% leakage of Calcein from Calcein-loaded liposomes at 4× MIC (Figure 7E). Moreover, SEM assay demonstrated that untreated cells exhibited unique shapes (short and slightly curved rods). The bottom images show the intact surface of *E. coli*, as seen clearly at higher magnification. However, treatment with MSI-1 (1× MBC) displayed obvious morphological changes as the cell walls were covered with substances resulting from serious disruptions in the surface of *E. coli*. The bottom images, at higher magnification, show that the cell walls were covered with substance resulting from serious disruptions in the surface of *E. coli* (Figure 7F). To more broadly examine whether MSI-1 affected bacterial envelope integrity, we determined the membrane permeability by calculating the percentage of O-nitrophenol production. After 2 h incubation, a concentration-dependent release of cytoplasmic β-galactosidase was observed (Figure 7G), reflecting that the activities in the supernatants of MSI-1 treated cells were 36.7% and 80.7% at peptide doses of 2× and 4× MIC, respectively, similar to that of cells treated with mellitin (49.9% and 98.5%, respectively). Thus, MSI-1 exerted a strong electrostatic attraction to the negative charges of LPS and aggregated on the bacterial surface after charge neutralization and then exhibited significant membrane rupture properties that contributed to its excellent antimicrobial activity (Figure 7H).

### MSI-1 at sub-MIC levels impacted the biological function of bacteria by binding DNA

**Figure 8:**
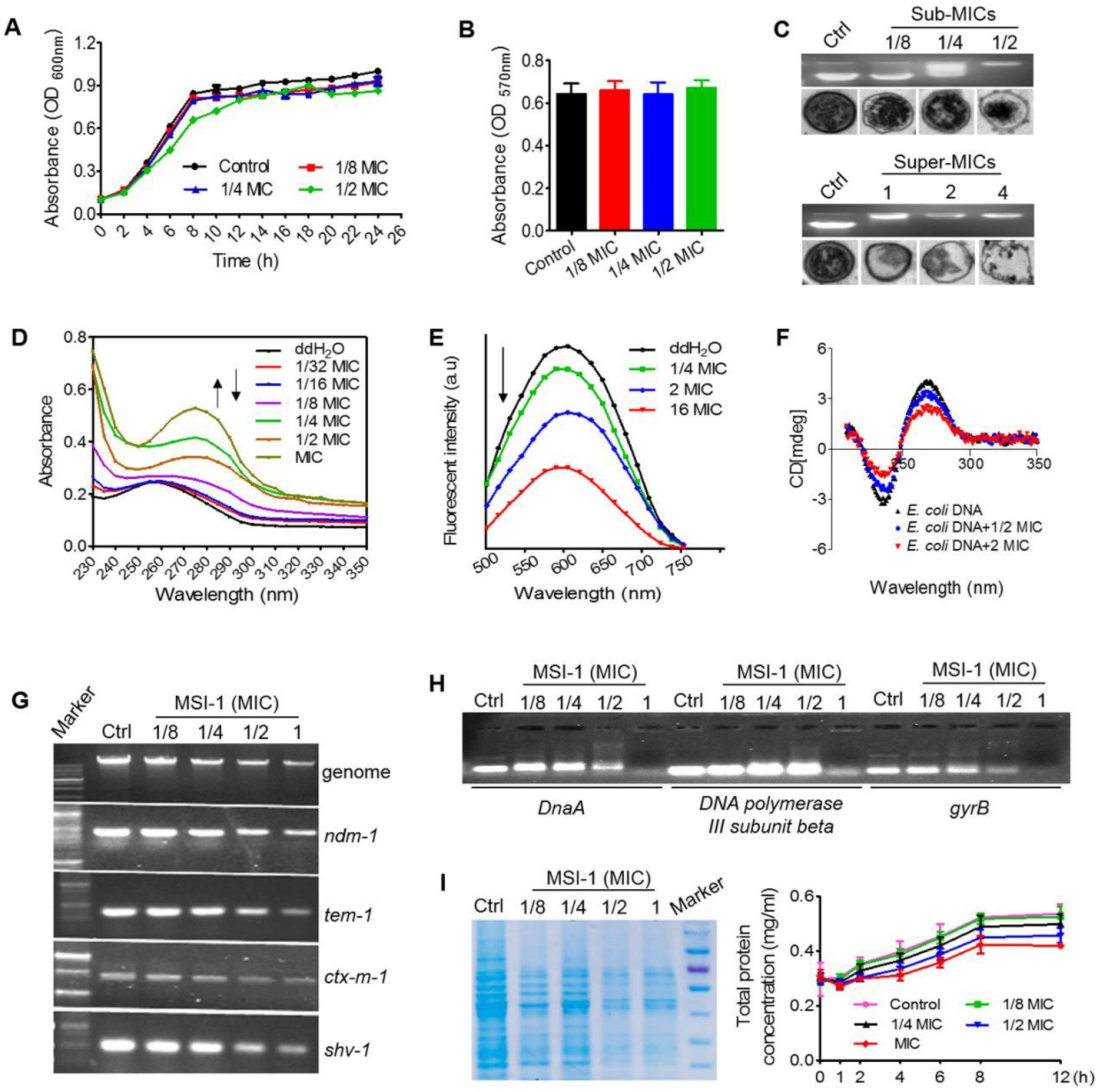
** The potential antibacterial mechanisms on bacterial DNA.** Growth curves **(A)** and bacteria viability **(B)** of penicillin-resistant *E. coli*. **(C)** Gel retardation assay for DNA binding and TEM assay for intracellular changes in *E. coli* morphology. Ctrl: PBS. **(D)** UV spectra of bacterial genomic DNA. **(E)** Competitive binding of MSI-1 and EB with bacterial DNA. **(F)** CD analysis for secondary structure change of *E. coli* genomic DNA after binding to peptide. The final spectra were the average of 5 scans. **(G)** Agarose gel electrophoresis for the replication of bacterial genomic DNA and β-lactamase-encoding genes in β-lactamase-producing drug-resistant bacteria.** (H)** Gel retardation electrophoresis for the interaction between *E. coli* DNA synthesis genes (*DnaA*, *DNA polymerase III subunit beta* and *gyrB*) and MSI-1.** (I)** Effect of MSI-1 on bacterioprotein synthesis. SDS-PAGE (left) and BCA (right) assays for the quantification of total bacterial proteins after sub-MICs peptide treatment for 8 h.

As shown in Figure 8A, the growth pattern of bacteria was not significantly altered when treated with MSI-1 at 1/4 and 1/8 MIC, while it exhibited a slight lag phase and lower absorbance during 4 to 12 h when treated with MSI-1 at 1/2 MIC. At the end of 26 h, the mean absorbance of the 1/2 MIC group did not significantly differ from that of the nontreated control. Similarly, the MTT assay also revealed that there was no significant difference in the cell viability of the control and peptide-treated *E. coli* within 26 h (p>0.05, Figure 8B). However, MSI-1 bound to* E. coli* genomic DNA at a dose of 1/4× MIC (2 μg/ml), showing as the disseminated migration trajectory because only a fraction of the genomic DNA could still be able to migrate into the gel in the same way as non-complexed DNA. And the electrophoretic movement of DNA was completely inhibited when the peptide concentration was higher than 4 μg/ml, accompanying with an obvious decrease in fluorescence intensity of the DNA-SYBR Green I complex. Moreover, unlike the ribosome-rich cytoplasm surrounding the DNA-containing nucleoid in the control, MSI-1 altered the native organization of the intracellular compartment by the appearance of asymmetrical regions of differing electron densities (Figure 8C). Interestingly, as the peptide concentrations increased (≥ MIC and ≤ 4× MIC), ribosomes were depleted from the cytoplasm, and the condensation of DNA that appeared in the control was diminished or abolished; however, the bacterial membrane still appeared intact. It should be noted that the cytoplasmic membrane ruptured was accompanied by cytoplasmic leakage when MSI-1 was administered at a dose of 4× MIC (32 μg/ml). These findings suggest that MSI-1 may also exert its activity by binding to bacterial DNA.

As shown in Figure 8D, after titrating MSI-1 into *E. coli* genomic DNA, hyperchromic effects were observed with the increase in peptide concentration, which could be resulted from the destabilization of the base pairs of bacterial DNA because increased peptide gradually padded the groove of DNA followed by the exposure of amino groups from the side chains of peptide to the aromatic bases of DNA 33. Meanwhile, MSI-1 contained phenylalanine and tryptophan; thus, the aromatic groups in MSI-1 peptide chain ending into the groove of bacterial DNA may lead to a slight red shift in the position of DNA's absorption peak from 260 nm to 275 nm 34. Additionally, we found that EB-DNA mixture fluorescence declined as the MSI-1 concentration increased, indicating that MSI-1 competed with EB in binding to *E. coli* genomic DNA, and some of the EB molecules that previously intercalated into the DNA base pairs were replaced by MSI-1 (see Figure 8E). That is, the decrease in the fluorescence intensity of DNA-EB complex was attributed to the formation of non-fluorescence complex DNA-EB-peptide. Overall, in addition to electrostatic interactions between the positive charges of peptides and the negative charges from phosphate groups of bacterial DNA, a groove-binding mode between the aromatic groups from peptide chain and genomic DNA may also exist. Additionally, MSI-1 also could intercalate between base pairs of double-stranded DNA like ethidium bromide. Moreover, CD measurements were taken to assess conformational changes of *E. coli* genomic DNA under peptide-DNA interaction. As shown in Figure 8F, free DNA had a typical positive band at 270 nm due to base stacking and a negative band at 240 nm because of the polynucleotide helicity of the B-DNA conformation 35. Once peptide was added, MSI-1 binding weakened the base-stacking interactions and decrease the positive band of DNA at 270 nm, but the binding tightened the double helix of DNA, resulting in the increase in the negative band of DNA at 240 nm. This finding indicates that MSI-1 induces the secondary structure change in bacterial genomic DNA.

In addition to inhibiting the expression of* E. coli* genomic DNA, MSI-1 also suppressed the replication of β lactamase-encoding genes, such as *ndm-1*, *ctx-m1, tem-1* and *shv-1*, which were cloned into the recombinant plasmid (Figure 8G). Moreover, we performed gel electrophoresis to detect the interaction of DNA synthesis-related genes with MSI-1 and ultimately confirmed that MSI-1 at sub-MIC also bound to the DNA synthesis-related genes to interfere the processes of DNA synthesis in *E. coli* (Figure 8H). At the protein level, after 8 h of treatment with MSI-1, the bacterial proteins decreased in a dose-dependent manner, and there was a significant difference between the 1/2× MIC MSI-1-treated group and the untreated control group (p ≤ 0.05) (Figure 8I). Therefore, at the sub-MIC level, MSI-1 disturbs the biological function of bacteria by binding to genomic DNA or DNA synthesis-related genes.

## Discussion

In the present study, the truncated peptide MSI had a significantly decreased amino acid sequence, cationic charges, antibacterial spectrum and antibacterial activity. Its lowered activity may be attributed to low cationic charges, amphiphilicity and α-helicity, which are key factors for AMP activity. Although, it has been reported that the increased number of positively charged amino acids in cationic AMPs was directly correlated with enhanced antibacterial activity 36, MSI-1 and MSI-3, which was modified by substituting Gly^3^ and Gly^13^ with Trp based on MSI, had lower positive charges but stronger antibacterial activity against various bacteria than MSI-78. Because in addition to electrostatic attraction, the α-helical content and hydrophobic force between the hydrophobic tail of phospholipids and the hydrophobic chain of AMPs are all important parameters for AMP activity 37. Here, an increase in percent helicity and amphiphilicity were detected in MSI-1 and MSI-3. Notably, compared with MSI-1, MSI-3 displayed a stronger activity attributed to the higher amphiphilicity despite its net charges being lower (+6 *vs* +5) after the truncation of a Lys residue. MSI-2, with the lowest amphiphilicity among peptide analogs, had the poorest antibacterial activity. This suggests that amphiphilicity is more important for the antibacterial activity of AMPs and the Trp introduction is a strategy for AMP design.

Subsequently, we confirmed that three mutants with potent antibacterial activity all exhibited low hemolysis activity (HC_50_ > 400 μM) against sRBCs and low cytotoxicity (IC_50_ > 40 μM) against murine spleen cells and other human eukaryotic cells (HaCaT, GES-1 and L02) compared with melittin, which has a rapid cytolytic action and destabilizes membranes of different cell types 28. Because the aggregation of MSI-1 or MSI-3 on HaCaT cells was much lower than that of melittin (Figure 3D-E). Importantly, MSI-3 with higher hydrophobicity possessed a relatively higher hemolysis rate and cytotoxicity than MSI-1, and this phenomenon was further confirmed by *in vivo* toxicity experiments. Therefore, there exists an optimum range of hydrophobicity ratios, and too high will increase the hemolytic activity of AMPs, while too low will reduce the activity of AMPs 38. The potent antibacterial activity and rapid bactericidal efficiency of MIS-1 against various bacteria, including drug-resistant bacteria, was identified. For acute systemic infection and chronic local infection of scalded skin induced by *E. coli*, it could improve animal survival rate or wound closure by eliminating bacterial counts in organs or subeschar to inhibit the systemic dissemination of bacteria, decreasing the excessive secretion of proinflammatory factors and then inhibiting the bacteria-induced inflammatory response to alleviate lung injury or reduce purulence production. Thus, the amino acid composition and arrangement of MSI-1 are characterized by superior antibacterial activity and low toxicity *in vivo/vitro*.

Potent broad-spectrum antimicrobial activity, high stability, and no antimicrobial resistance are the advantages of AMPs over other antibiotics 3. The antimicrobial resistance to peptides has been reported one after another currently 39. Excitingly, MSI-1 circumvents drug resistance production after 15 subcultures following the initial exposure. Some factors, such as chemical, biological, physical and physiological conditions may affect the antimicrobial activity of AMPs 17-19, 29. In this study, MSI-1 displayed perfect pH tolerance even under the extreme pH values of 3 and 11, significant high salt resistance (400 mM), and could overcome the influence of temperature variations (from -80 °C to 100 °C). After pepsin treatment, the peak time of MSI-1 were prolonged because of the production of the peptide hydrolysates. Interestingly, the activities of the peptide hydrolysates were still retained (Fig. 4B), which may attribute to the retaining of the active amino acid sequence. However, the structures and the relationship between amino acid sequence and activity of the peptide hydrolysates still need further study. Although either mouse plasma or serum could degrade MSI-1 and weaken its antibacterial activity, there were still 60% remaining peptide and approximately 50% bacteriostatic activity post 2 h coincubation, indicating its potential for systemic therapeutic applications.

The binding of cationic AMPs to the cell membrane is crucial to its biological activity. MSI-1 could bind to LPS, a major component of *E. coli* membrane, and neutralize the negative charges on the *E. coli* surface through electrostatic attraction and then aggregate on bacteria. Alpha-helical peptides, such as membrane lysis AMPs, have obvious hydrophobic and hydrophilic surfaces that favor the insertion of AMPs into bacterial cell membranes and subsequent membrane permeability enhancement and membrane damage 40. MSI-1 exhibited a typical α-helix configuration in LPS, MLVs and SDS (Figure 1D) with α-helical contents of 31.4%, 34.2% and 97.5%, respectively. It had a destructive effect on the bacterial cell membrane in a dose-dependent manner, reflecting by the increased PI uptake, cytoplasmic β-galactosidase release and Calcein leakage and the ultimate morphological changes of *E. coli* after MSI-1 treatment.

At sub-MIC levels, MSI-1 could not interfere with the basic viability of* E. coli* but bound to bacterial genomic DNA, as shown by the complete inhibition of the electrophoretic movement of the DNA in gel retardation assay, the asymmetrical regions of differing electron densities of DNA, and even the depletion of DNA when peptide concentration was higher than 4 μg/ml. Because of the favorable electrostatic potential and amphipathic hydrophobicity of cationic AMPs they cause aggregation of ribosomes which are polyanionic, and mediate this interaction in bacteria 41. In addition to electrostatic interactions between the positive charges of MSI-1 and the negative charges from phosphate groups of *E. coli* DNA, the groove-binding mode between this peptide and *E. coli* genomic DNA may also exist. Because the hyperchromic effects and a slight redshift in the position of DNA absorption peak from 260 to 275 nm in UV spectroscopic assay, and declined fluorescence of the EB-DNA mixture in EB competitive binding experiment were observed. Peptide-DNA interactions are important in replication, transcription, repair, and epigenetic modifications of DNA. In this study, the binding of MSI-1 to DNA induced conformational changes of *E. coli* genomic DNA because MSI-1 may weaken the base-stacking interaction and tighten the double helix of DNA. Subsequently, this peptide-DNA interaction could inhibit the expression of genomic DNA and the replication of β-lactamase-encoding genes. Moreover, the binding of MSI-1 to DNA synthesis-related genes, such as* DnaA*, *DNA polymerase III subunit beta* and *gyrB*, could interfere with the processes of DNA synthesis and bacterial protein expression.

In conclusion, we obtained a 14-mer peptide MSI-1 with desirable selectivity of action against bacteria and stability against violent pH, temperature variations and high NaCl concentrations. Moreover, the amino acid composition and arrangement gives the peptide a much lower cost and enhanced antibacterial/bactericidal activities against various G^+^ and G^-^ bacteria, even penicillin-resistant bacteria, and a low potential for the emergence of resistance. *In vivo*, this peptide exhibits a potent protective effect against drug-resistant *E. coli-*induced mice with acute bacterial peritonitis or local skin infection after scald. The possible mechanisms underlying its mode of action involve direct antimicrobial activity *via* significant membrane rupture properties at the super-MIC level and the dysfunction of bacterial biological function by binding to bacterial DNA at the sub-MIC level. MSI-1, thus, could be a potential candidate for drug development against infection induced by drug-resistant bacteria.

## Figures and Tables

**Table 1:** MICs (μg/ml) of the designed peptides against several bacterial strains.

	Microorganisms	MIC (μg/ml)						
		MSI-1	MSI-2	MSI-3	MSI-4	MSI	MSI-78	Penicillin
G^-^	*E. coli* ATCC25922	8	64	4	16	128	16	0.25
	*E. coli* A2 (ESBLs ^(+)^)	8	128	8	32	>256	8	>256
	*E. coli* A5 (ESBLs ^(+)^)	4	128	4	16	128	16	>256
	*E. coli* A9 (ESBLs ^(+)^)	8	64	8	32	64	16	>256
	*P. aeruginosa* ATCC27853	16	128	16	32	256	16	0.5
	*P. aeruginosa* T1 (ESBLs ^(+)^)	16	>256	16	16	>256	32	>256
	*P. aeruginosa* T6 (ESBLs ^(+)^)	8	>256	8	32	256	16	>256
	*P. aeruginosa* T8 (ESBLs ^(+)^)	16	>256	8	64	>256	16	>256
	*K. peneumoniae* ATCC4352	4	256	8	32	>256	8	1.0
	*K. peneumoniae* B4 (ESBLs ^(+)^)	8	>256	16	64	>256	16	>256
	*K. peneumoniae* B6 (ESBLs ^(+)^)	8	128	8	32	128	16	>256
	*K. peneumoniae* B9 (ESBLs ^(+)^)	16	>256	8	16	256	32	>256
	*E. cloacae* S1 (AmpC ^(+)^)	4	128	4	16	>256	16	>256
	*E. cloacae* S3 (AmpC ^(+)^)	8	>256	4	32	128	16	>256
	*Shigella flexneri*	8	>256	8	16	256	32	>256
								
G^+^	*S. aureus* ATCC25923	16	256	8	16	64	16	0.125
	MSSA D1	8	128	4	32	128	16	0.5
	MSSA D4	8	>256	8	16	>256	32	0.125
	MRSA M101	16	>256	16	32	>256	32	>256
	MRSA M108	8	>256	8	64	>256	16	>256
	*S. epidermidis* ATCC35984	8	128	4	16	128	16	0.5
	MSSE E8	8	256	8	16	128	16	1.0
	MSSE E3	16	>256	8	16	>256	32	2.0
	MRSE N109	16	>256	16	32	>256	16	>256
	MRSE N104	16	>256	16	32	>256	32	>256
	*S. pneumoniae* ATCC 49619	8	256	8	16	256	16	0.25
	PISP F22	16	>256	16	32	>256	16	1.0
	PISP F34	8	128	8	16	>256	32	0.5
	PRSP P112	32	>256	16	32	128	32	>256
	PRSP P105	16	>256	16	64	>256	16	>256
	*E. faecium*	4	>256	4	16	>256	8	>256
	*E. faecalis*	8	>256	8	32	>256	16	>256

MSSA: Methicillin sensitive *Staphylococcus aureus*; MRSA: Methicillin resistant *Staphylococcus aureus*; MSSE: Methicillin sensitive *Staphylococcus epidermidis*; MRSE: Methicillin resistant *Staphylococcus epidermidis*; PISP: Penicillin international susceptible *Streptococcus pneumoniae*; PRSP: Penicillin resistant *Streptococcus pneumoniae*. Clinical strains with different numbers were isolated from human clinical specimens by the Medical Laboratory Center of Zhongda Hospital (Nanjing, Jiangsu, China).

**Table 2:** MIC and MBC of MSI-1 against drug-resistant bacteria.

Microorganism strains		MSI-1 (µg/ml)			Melittin (µg/ml)	
		MIC	MBC		MIC	MBC
G^-^						
	*E. coli* ^a^	4	16		4	8
	*P. aeruginosa* ^a^	8	32		4	8
	*Helicobacterpylori* SS1	32	64		8	16
	*E. coli* BL21(DE3)*-*NDM-1^b^	8	8		4	8
	*K. pneumonia* ^a^	16	32		8	16
						
G^+^	*S. aureus* ^c^	8	16		8	32
	*S. epidermidis* ^c^	8	16		4	16
	*S. pneumoniae* ^c^	8	32		8	16
	*S. pyogenes* ^d^	16	32		8	16
	MRSA ^e^	8	16		4	8
						

^ a^ Penicillin-, Imipenem- and Ceftazidime- resistant clinical isolates. ^b^ *ndm-1*-carrying recombinant *E. coli* 33 ^ c^ Amoxicillin-, Cephalexin- and Levofloxacin- resistant clinical isolates. ^ d^ Penicillin-, Erythromycin- and Levofloxacin- resistant clinical isolates. ^ e^ Methicillin-resistant clinical isolates.

**Table 3:** Primers for PCR assay.

Primer Name	Sequence (5' to 3')
*DnaA*	(F) 5'-AGC AGT CCA TTG ATA TTA TTA AGG-3'
	(R) 5'-GAT GAG TTA CCA GCC ACA G-3'
*DNA polymerase* *III subunit beta*	(F) 5'-GTG CCA CCA TTT CCA TCT C-3'
	(R) 5'-TCC TAC GCT ACC GAT TCT C-3'
*gyrB*	(F) 5'-TCC GAA CTG TAC CTT GTG-3'
	(R) 5'-ATG ATG ATG CTG TGA TAA CG-3'
*ndm-1*	(F) 5'-CGC GGA TCC ATG GAA TTG CCC AAT ATT ATG CAC CCG G-3'
	(R) 5'-CCG GAA TTC TCA GCG CAG CTT GTC GGC CAT-3'
*blaTEM-1*	(F) 5'-ATG AGT ATT CAA CAT TTT CGT G-3'
	(R) 5'-TTA CCA ATG CTT AAT CAG TGA G-3'
*blaSHV-1*	(F) 5'-ATG CGT TAT ATT CGC CTG TG-3'
	(R) 5'-TTA GCG TTG CCA CTG GTG CTC G-3'
*blaCTX-M1*	(F) 5'-GCT GTT GTT AGG AAG TGT GC-3'
	(R) 5'-CCA TTG CCC GAG GTG AAG-3'

## Abbreviations

AMPs: antimicrobial peptides
Gly: glycine
Arg: arginine
Trp: tryptophan
Lys: Lysine
Ala: alanine
Val: valine
MIC: minimum inhibitory concentration
MBC: minimal bactericidal concentration
IPTG: Isopropyl β-D-Thiogalactoside
MHB: Mueller-Hinton broth
CFUs: colony forming units
DAPI: 4',6-diamidino-2-phenylindole
G^+^ bacteria: Gram-positive bacteria
G^-^ bacteria: Gram-negative bacteria
NDM-1: New Delhi metallo-beta-lactamase-1
MTT: 3-(4,5-Dimethylthiazol-2-yl)-2,5-diphenyltetrazolium bromide
TNF-α: tumor necrosis factor-α
IL-6: interleukin 6
PBS: phosphate buffer saline
TFA: trifluoroacetic acid
DPPG: 1,2-Dipalmitoyl-sn-glycero-3-phospho-(1'-rac-glycerol)
SDS: sodium dodecyl sulphate
DPPE: 1, 2-Dipalmitoyl-sn-glycero-3-phosphoethanolamine
MLVs: multilamellar vesicles
VEGF: vascular endothelial growth factor
LPS: lipopolysaccharide
PI: propidium iodide
DPPE: 1, 2-Dipalmitoyl-sn-glycero-3-phosphoethanolamine
EGF: epidermal growth factor
H&E: haematoxylin and eosin
HC_50_: 50% hemolysis concentration
SDS-PAGE: sodium dodecyl sulphate-polyacrylamide gel electrophoresis
CMC-Na: sodium carboxyl methyl cellulose
CD: Circular dichroism
ONPG: o-nitrophenyl-β-d-galactopyranoside
MW: molecular weight
